# Recent Advances in Liver Cancer Stem Cells: Non-coding RNAs, Oncogenes and Oncoproteins

**DOI:** 10.3389/fcell.2020.548335

**Published:** 2020-10-07

**Authors:** Juan Li, Ying Zhu

**Affiliations:** ^1^Department of Radiotherapy Oncology, The Second Affiliated Hospital of Dalian Medical University, Dalian, China; ^2^Department of Infectious Disease, The First Affiliated Hospital of Dalian Medical University, Dalian, China; ^3^Liver Disease Center of Integrated Traditional and Western Medicine, Institute of Integrative Medicine, Dalian Medical University, Dalian, China

**Keywords:** hepatocellular carcinoma, liver cancer stem cells, non-coding RNAs, oncogenes, oncoproteins

## Abstract

Hepatocellular carcinoma (HCC) is one of the most prevalent malignancies worldwide, with high morbidity, relapse, metastasis and mortality rates. Although liver surgical resection, transplantation, chemotherapy, radiotherapy and some molecular targeted therapeutics may prolong the survival of HCC patients to a certain degree, the curative effect is still poor, primarily because of tumor recurrence and the drug resistance of HCC cells. Liver cancer stem cells (LCSCs), also known as liver tumor-initiating cells, represent one small subset of cancer cells that are responsible for disease recurrence, drug resistance and death. Therefore, understanding the regulatory mechanism of LCSCs in HCC is of vital importance. Thus, new studies that present gene regulation strategies to control LCSC differentiation and replication are under development. In this review, we provide an update on the latest advances in experimental studies on non-coding RNAs (ncRNAs), oncogenes and oncoproteins. All the articles addressed the crosstalk between different ncRNAs, oncogenes and oncoproteins, as well as their upstream and downstream products targeting LCSCs. In this review, we summarize three pathways, the Wnt/β-catenin signaling pathway, phosphatidylinositol 3-kinase (PI3K)/protein kinase B (Akt) signaling pathway, and interleukin 6/Janus kinase 2/signal transducer and activator of transcription 3 (IL6/JAK2/STAT3) signaling pathway, and their targeting gene, c-Myc. Furthermore, we conclude that octamer 4 (OCT4) and Nanog are two important functional genes that play a pivotal role in LCSC regulation and HCC prognosis.

## Introduction

Among tumor types, liver cancer is the third leading cause of death in humans around the globe (Torre et al., 2015; Forner et al., 2018). Hepatocellular carcinoma (HCC) is one of the most common subclass accounting for 90% of liver cancer (Bruix et al., 2014). Most HCC patients are no longer eligible for curative treatment, such as transplantation or surgical resection, because of disease progression to the late stage. Simultaneously, while molecular targeted therapies and chemotherapy are available for partial HCC patients, clinical benefits remain unsatisfactory. As a result, exploration of new systemic treatment approaches for HCC is important due to poor outcomes (Blum, 2005; Forner et al., 2012). Interestingly, using surface markers, studies have identified cancer stem cells (CSCs) and isolated CSC subpopulations from HCC cells in the field of liver CSCs (LCSCs) (Liu Y.M. et al., 2015). Although LCSCs only represent a small subset of liver cancer cells, they are considered to be responsible for HCC tumorigenesis, progression, metastasis and recurrence. Therefore, many scholars have conducted studies on LCSCs. In addition, scholars have summarized their results from the perspective of genes or RNA. However, new research results are constantly emerging. To provide LCSC researchers with more information, this review will summarize studies newly reported from the perspective of ncRNAs, oncogenes and oncoproteins.

## The Role and Classification of ncRNAs

The main function of RNA is to bridge the transformation process from genetic information to translation of genetic information into proteins. In transcriptional precursor RNA, more than 70% of the genome is transcribed into non-coding RNAs (ncRNAs), only approximately 20% of which are transcribed into messenger RNA (mRNAs). The common feature of ncRNAs is that they can be transcribed from the genome but perform their respective biological functions at the RNA level without being translated into proteins. There is sufficient evidence to demonstrate the important role of ncRNAs in regulating LCSCs. ncRNAs include microRNAs (miRNAs), long ncRNAs (lncRNAs), small interfering RNAs (siRNAs), ribosomal RNAs (rRNAs), transfer RNAs (tRNAs), and small nuclear RNAs (snRNAs). Here, we will focus on the most studied members of the ncRNA family, miRNAs and lncRNAs.

### miRNAs Associated With LCSCs

MicroRNA is a subset of the ncRNA family that can regulate expression of more than 60% of human genes. While miRNA is a group of short ncRNAs containing approximately 22 nucleotides and are not completely complementary to target mRNAs, they inhibit post-transcriptional translation by binding to the 3′-untranslated region (3′-UTR) of target mRNAs (DeSano and Xu, 2009; Gargalionis and Basdra, 2013). Moreover, dysregulation of miRNA expression is linked to tumorigenesis in humans (Budhu et al., 2008; Ji et al., 2009a; Li et al., 2010; Fu et al., 2014) and regulates the stemness features of CSCs (Ji et al., 2009b). The characteristics of several miRNAs whose expression is associated with LCSCs are well known, such as miR-130b (Ma et al., 2010), miR-21 (Tomimaru et al., 2010; Zhou et al., 2013), miR-214 (Xia et al., 2012), miR-425-3p (Vaira et al., 2015), and miR-517a (Toffanin et al., 2011). Here, we summarize the recently identified miRNAs whose deregulation enhances or suppresses LCSC properties (Supplementary Figure 1).

#### miRNAs That Enhance LCSC Properties

##### miR-429

E2F transcription factor 1 (E2F1) has been found to be a novel regulator of pluripotent stem cells (Yeo et al., 2011). Interestingly, the protein-protein interaction between E2F1 and RB transcriptional co-repressor 1 (RB1) was significantly weakened upon transfection with miR-429. Moreover, miR-429 can modulate the transcriptional activity of E2F1 via direct targeting of RB binding protein 4 (RBBP4). Furthermore, the stemness-related gene OCT4 was identified as an E2F1-responsive gene and was upregulated upon RBBP4 silencing or high miR-429 expression. In sum, high expression of miR-429 contributed to self-renewal, tumorigenicity, proliferation and chemoresistance in HCC. In addition, miR-429 was found to target a novel functional axis, RBBP4/E2F1/OCT4, to manipulate HCC (Li L. et al., 2015).

##### miR-1246

Glycogen synthase kinase 3β (GSK3β) and axis inhibition protein 2 (AXIN2) are negative regulators of Wnt signaling and are tumor suppressors in HCC (Reya and Clevers, 2005). A recent study demonstrated that miR-1246 promotes tumorigenesis, metastasis and chemoresistance of LCSCs by activating the Wnt/β-catenin signaling pathway. Mechanistically, an *in silico* prediction indicated that AXIN2 and GSK3β were potential downstream targets of miR-1246. Interestingly, miR-1246 activated the Wnt/β-catenin pathway by suppressing GSK3β and AXIN2 expression, which are key members of the β-catenin destruction complex. Furthermore, OCT4 was the direct upstream regulator of miR-1246, which activated miR-1246 expression through miR-1246 promoter binding and cooperatively drove β-catenin activation in LCSCs (Chai et al., 2016).

##### miR24-2

miR24-2 can promote tumorigenesis by epigenetically enhancing the tyrosine kinase Src and can epigenetically regulate liver cancer by altering the expression of various Histone H3/4 epigenetic modifications in LCSCs. Moreover, histone deacetylase 3 (HDAC3), Nanog and PI3K were found to be key players in the signaling pathways mediated by miR24-2. Furthermore, miR24-2 targeted the protein arginine methyltransferase 7 (PRMT7) 3′-UTR and inhibited PRMT7 expression, thereby reducing the bi/trimethylation of histone H4R3. Importantly, miR24-2 promoted the transcriptional activity and maturation of the miR675 precursor (pri-miR675) through binding to Nanog in LCSCs. lncRNA HULC plays a key role in the carcinogenesis triggered by miR24-2. Moreover, miR24-2-dependent PI3K activation promoted autophagy (Wang L. et al., 2019).

##### miR-199a-3p, miR-155

Transforming growth factor beta 1 (TGF-β1) has been confirmed to be an important enhancer of CSCs and epithelial-mesenchymal transition (EMT) (Polyak and Weinberg, 2009). miR-199a-3p plays an important role and is upregulated in LCSCs. Consistently, overexpression of TGF-β1 and hepatitis B virus X (HBx) have been associated with LCSC properties and poor prognosis in hepatitis B virus (HBV)-related liver cancer. TGF-β1 cooperation with HBx can activate the c-Jun N-terminal kinase (JNK)/c-Jun pathway, while miR-199a-3p, a regulator of hepatic progenitor cell (HPC) transformation, can be activated by c-Jun. In conclusion, TGF-β1/HBx co-regulated the miR-199a-3p signaling axis targeting malignant transformation of HPCs (Dong et al., 2019). Furthermore, miR-155 overexpression promoted cell EMT in liver cancer cells, and overexpression of miR-155 promoted the stemness of LCSCs via down-regulation of tumor protein P53 inducible nuclear protein 1 (TP53INP1), which is a downstream target gene of miR-155. In addition, in vitro, TGF-β1 indirectly downregulated TP53INP1 expression via miR-155 upregulation in liver cancer cells (Ji et al., 2015; Liu Y.M. et al., 2015; Liu et al., 2015a,b).

##### miR-500a-3p and miR-589-5p

Evidence has indicated that the JAK/STAT signaling pathway acts as a critical regulator in several well-known CSCs (Jove, 2000). One study reported that miR-500a-3p promotes CSC properties by targeting suppressor of cytokine signaling (SOCS)2, SOCS4 and protein tyrosine phosphatase non-receptor type 11 (PTPN11) through STAT3 signaling activation (Jiang et al., 2017). Another study found that overexpression of miR-589-5p decreased overall and relapse-free survival in HCC. Further mechanistic analysis revealed that miR-589-5p activated the STAT3 pathway by inhibiting its negative regulators. Moreover, upregulation of miR-589-5p enhanced LCSC properties (Long et al., 2018).

Furthermore, zinc finger e-box binding homeobox (ZEB)1/2 is a key transcription factor in EMT, and is the most prominent target of the miR-200 family (Burk et al., 2008). Deregulation of miR-200b was involved in regulation of LCSCs, the miR-200b–ZEB1 circuit was found to regulate diverse LCSCs (Tsai et al., 2017), and miR-219 down-regulated E-cadherin via its mRNA 3′UTR, thus playing a role in the sensitivity of HCCs to sorafenib (Si et al., 2019), miR-137 expression was upregulated in CD44-positive CSCs and found to be associated with a significantly shorter survival periods for HCC patients (Sakabe et al., 2017).

#### miRNAs That Suppress LCSC Properties

##### miR-125b

Increasing evidence suggests that EMT contributes to metastasis and recurrence in HCC (Choi and Diehl, 2009). Zhou et al. (2015) found that overexpression of miR-125b could attenuate migration, chemoresistance and LCSC generation by suppressing EMT. Moreover, they revealed that miR-125b suppressed EMT by targeting small mothers against decapentaplegic (SMAD)2 and SMAD4. These findings suggest that ectopic expression of miR-125b is a potential HCC treatment strategy (Zhou et al., 2015).

##### miR-192-5p

miR-192-5p was found to be significantly down-regulated in LCSCs. Suppression of miR-192-5p markedly increased LCSC numbers and the features of LCSCs through targeting of poly(A) binding protein cytoplasmic 4 (PABPC4). The axis of tumor protein p53 (TP53) mutation/mir-192 promoter hypermethylation/reduced miR-192-5p/increased PABPC4 was identified in HCCs expressing high levels of CSC markers. These findings reveal a genetic regulatory signaling pathway shared by different LCSCs (Gu et al., 2019).

##### miR-302a/d

miR-302a/d negatively regulates spheroid formation and cell growth and promotes apoptosis of liver cancer cells by suppressing the targeted E2F transcription factor 7 (E2F7) gene. In one study, miR-302a/d inhibited LCSC cell cycle entry and self-renewal via targeting the E2F7/Akt axis. These results suggest that miR-302a/d and E2F7 might be potential biomarkers of LCSCs (Ma et al., 2018).

##### miR-26b-5p

Epithelial cell adhesion molecule (EpCAM) is one of the most prevalent LCSC markers. Recently, researchers reported that miR-26b-5p targets both heat shock protein family A member 8 (HSPA8) and EpCAM. Reduced expression of miR-26b-5p enhanced LCSC invasion, migration and tumorigenesis. Moreover, miR-26b-5p was responsible for maintaining EpCAM-positive LCSCs by targeting of HSPA8 (Khosla et al., 2019).

Furthermore, miR-1305 overexpression reversed the suppressor that inhibited LCSC properties by suppressing the ubiquitin-conjugating enzyme E2T (UBE2T)-dependent Akt-signaling pathway (Wei et al., 2019). While knockdown of miR-25 enhanced the sensitivity of LCSCs to TNF-related apoptosis inducing ligand (TRAIL)-mediated apoptosis via the phosphatase and tensin homologue (PTEN)/PI3K/Akt/Bad signaling pathway (Feng et al., 2016). miR-365 directly regulated Ras-related C3 botulinum toxin substrate 1 (RAC1) by binding with the mRNA 3′UTR and affected HCC drug resistance (Jiang et al., 2019). In addition, miR-486 directly targeted sirtuin1, which exhibits high expression in self-renewing and tumorigenic LCSCs (Yan et al., 2019).

### LncRNAs Associated With LCSCs

Long ncRNAs are a subclass of ncRNAs longer than 200 nucleotides. They have emerged as critical epigenetic regulators of gene expression and share some characteristics of mRNAs (Devaux et al., 2015). lncRNAs exert their functions via diverse mechanisms, including cytoplasmic complexes, modulation of gene expression, nuclear scaffolding, transcriptional regulation and pairing with other RNAs (Ulitsky and Bartel, 2013). lncRNAs can regulate gene expression through chromosome remodeling, transcription and post-transcriptional processing. Dysregulation of lncRNA expression has been associated with widespread development of many cancers (Zhang M. et al., 2016; Xiaoguang et al., 2017). We summarize the latest deregulated lncRNAs that enhance or suppress LCSC properties (Supplementary Figure 2).

#### LncRNAs That Enhance LCSC Properties

##### lncTCF7 and lnc-β-Catm

lncTCF7 can regulate transcription factor 7 (TCF7) expression by recruiting the SWI/SNF complex in the nuclei of LCSCs. Then, the TCF7 expression triggers Wnt signaling to initiate self-renewal of LCSCs. In sum, lncTCF7-mediated Wnt signaling primes LCSC self-renewal and tumor propagation (Wang et al., 2015). In addition, a study revealed a new transcribed lncRNA called lncRNA β-catenin methylation (lnc-β-Catm), which could also regulate self-renewal of LCSCs. Moreover, lnc-β-Catm was responsible for inhibiting β-catenin ubiquitination, allowing β-catenin to activate Wnt–β-catenin signaling and sustaining the stemness of LCSCs (Zhu et al., 2016a).

##### DANCR

In one study, genome-wide analyses identified tumor-associated lncRNA-DANCR. Dysregulation of DANCR was explored in HCC tumorigenesis and colonization. The activation of DANCR was confirmed to be associated with poor survival of HCC patients. Recently, Yuan et al. (2016) reported that lncRNA-DANCR was overexpressed in LCSCs. Experiments showed that knockdown of DANCR decreased stem-cell properties and tumor cell vitality. In further mechanistic studies, DANCR associated with Catenin Beta 1 blocked the repressive effect of miR-2214, miR-199a, and miR-320a (Yuan et al., 2016).

##### lncBRM

LINCR-0003 (lncBRM) was overexpressed in LCSCs and maintained their self-renewal stemness properties via Yes-associated protein 1 (YAP1) signaling. In addition, lncBRM associated with Brahma (BRM) initiated the BRM/SWI2-related gene 1 (BRG1)/BRM switch. Next, the BRG1-associated factor complex activated YAP1 signaling. Furthermore, lncBRM expression with the addition of YAP1 signaling was associated with the prognosis of HCC (Zhu et al., 2016b).

##### HAND2-AS1

INOsitol-requiring 80 (INO80) chromatin-remodeling complex, which is a conserved complex that modifies chromatin using the energy of adenosine triphosphate (ATP), controls gene expression and maintains stem cell properties (Ayala et al., 2018). One study revealed that lncRNA HAND2-AS1 expression was upregulated in LCSCs. Importantly, HAND2-AS1 recruited the INO80 complex to bone morphogenetic protein receptor type 1A (BMPR1A), inducing bone morphogenetic protein (BMP) signaling activation. Mechanistically, overexpression of lncRNA HAND2-AS1 associated with the INO80 complex can promote the self-renewal of LCSCs and drive liver oncogenesis (Wang et al., 2019b).

##### CUDR

Cancer upregulated drug resistant (CUDR) is a new ncRNA gene that is highly expressed in HCC. A study revealed that decreased phosphatase and tensin homolog (PTEN) might enhance the binding ability of CUDR to Cyclin D1. In this study, the CUDR-Cyclin D1 complex loaded onto the lncRNA H19 promoter region enhanced H19 expression. Moreover, the CUDR-Cyclin D1-CTC-binding factor (CTCF) complex promoted c-Myc expression (Pu et al., 2015). SET1A is a component of the histone methyltransferase complex. One study found that SET-domain-containing 1A (SET1A) cooperated with CUDR to promote malignant transformation of hepatocyte-like SCs (Li et al., 2016). Furthermore, research has shown that CUDR is highly upregulated in liver cancer and can cause abnormal β-catenin signaling during malignant transformation of LCSCs (Gui et al., 2015).

##### lncHOXA10

HOXA10 (homeobox A10) is a member of the HOX transcription factor family, which is highly expressed in liver tumors. HOXA10 interacts with some signaling pathways and participates in many types of cancer (Cui et al., 2014; Li et al., 2014). Recently, a study found that HOXA10 is upregulated during liver tumorigenesis and tumor-initiating cell (TIC) self-renewal. The authors found that both lncHOXA10 and HOXA10 were highly expressed and participated in self-renewal regulation in liver cancer and liver TICs. lncHOXA10 interacts with NURF chromatin remodeling complex and binds to the HOXA10 promoter to drive transcription initiation (Shao et al., 2018).

Furthermore, lncRNA HCG11 regulates insulin-like growth factor 2 mRNA-binding protein 1 (IGF2BP1) to inhibit apoptosis of HCCs via mitogen-activated protein kinase (MAPK) signaling transduction (Xu et al., 2017), and lncZic2 drives the self-renewal of liver TICs via the myristoylated alanine rich protein kinase C substrate (MARCKS) and MARCKS like 1 (MARCKSL1) (Chen et al., 2018). Moreover, lncRNA n339260 (Zhao et al., 2018) and lncCAMTA1 (Ding et al., 2016) were suggested to be new prognostic biomarkers of LCSCs.

#### LncRNAs That Suppress LCSC Properties

##### lnc-DILC

The suppressor lnc-DILC resides in both the nucleus and cytoplasm. A recent study showed the subcellular distribution of lnc-DILC and revealed its nuclear localization in LCSCs. Likewise, it was determined that lnc-DILC could depress IL-6 transcription and regulate LCSC expansion by suppressing IL-6 autocrine signaling. Interestingly, knockdown of lnc-DILC affected IL-6 transcription, STAT3 activation and LCSC expansion. Nuclear factor kappa B (NF-κB) was found to be an essential link between inflammation and cancer (Ben-Neriah and Karin, 2011) and to play a pivotal role in CSC maintenance (Kagoya et al., 2014). In another recent study, the authors clarified a paradigm of LCSC expansion in which lnc-DILC functions as a novel link connecting tumor necrosis factor (TNF)-a/NF-κB signaling with the autocrine IL-6/STAT3 cascade (Wang X. et al., 2016).

##### DLX6-AS1

lncRNA distal-less homeobox 6 antisense 1 (DLX6-AS1) belongs to the DLX gene family (Wang P. et al., 2017). One study demonstrated that DLX6-AS1 is highly expressed in HCC and serves as an oncogene targeting the DLX6-AS1/miR-203a/matrix metallopeptidase 2 (MMP-2) pathway (Zhang et al., 2017). Intriguingly, DLX6-AS1 can promote the stemness of osteosarcoma cells by regulating miR-129-5p/delta like non-canonical notch ligand 1 (DLK1) (Zhang et al., 2018). Additionally, cell adhesion molecule 1 (CADM1) expression was downregulated and facilitated tumorigenesis in HCC (Zhang W. et al., 2016). Another recent study showed that suppression of DLX6-AS1 inhibited tumorigenesis through the STAT3 signaling pathway, which restrains CADM1 promoter methylation in LCSCs (Wu et al., 2019).

## The Role of Oncogenes or Oncoproteins Associated With LCSCs

Current evidence indicates that during hepatocarcinogenesis, one potential pathogenic mechanism is abnormalities in oncogenes or oncoproteins. Interestingly, oncogenes play an important role in cell growth, proliferation and division (Hinds et al., 1989; Rochlitz et al., 1993). Genes with deletions, insertions or mutations may lose their functions and are related to cancer development. A large number of experiments have shown that abnormalities in oncogenes or the expression of oncoproteins are implicated in oncogenesis, tumor progression and metastasis by targeting LCSCs. According to GeneCards^1^, which shows the localization of human genes, gene subcellular locations will be described based on compartments as follows: nucleus, cytoplasm, and plasma membrane, among others (Supplementary Table 1).

### Oncogenes or Oncoproteins Mainly Located in the Nucleus of Human Cell

#### Sox9

The sex determining region Y box 9 (Sox9) protein is predominantly localized in the nucleus of HCCs. Sox9 is a transcription factor that is expressed in several cancers (Guo et al., 2012; Sarkar and Hochedlinger, 2013). Sox9 is significantly highly expressed in HCC and associated with decreased survival. Consistently, the proportion of Sox9 knockdown cells in S and G2/M phases was reduced and that in G0/G1 phase was increased. Furthermore, the expression of Sox9 was coincident with expression of the LCSC markers CD13 and OCT4. Knockdown of Sox9 expression in LCSCs cells resulted in a reduction in the expression of the stem cell transcription factors B cell-specific Moloney murine leukemia virus integration site 1 (BMI-1), OCT4 and Nanog, as well as in α-fetoprotein and β-catenin. Additionally, Sox9 was decreased during asymmetrical cell division and regulated the asymmetrical-to-symmetrical cell division switch in LCSCs (Liu C. et al., 2016).

#### MacroH2A1

Macrohistone H2A (MacroH2A) is a subclass of the H2A family containing two isoforms, encoded by macroH2A1 and macroH2A2. The MacroH2A1 gene is associated with tumorigenesis in many cancer types (Gaspar-Maia et al., 2013; Borghesan et al., 2016). Interestingly, macroH2A1 can protect differentiated HCC cells from chemotherapeutics as a marker (Rappa et al., 2013; Borghesan et al., 2016). A recent study found that downregulation of macroH2A1 enhanced the expression of stemness-related genes and hypoxia factor. Furthermore, depletion of macroH2A1 activated the phosphorylated nuclear factor kappa B p65 pathway, which is responsible for inducing LCSCs (Lo Re et al., 2018b). Knockdown of macroH2A1 led to LCSC-like features and massive alterations to the nuclear architecture in HCCs (Douet et al., 2017). MacroH2A1-depleted cells showed two changes in lipid metabolism and glucose in LCSCs: massive acetyl-coA upregulation, which transformed lipid content; and increased activation of the pentose phosphate pathway, which provides precursors for nucleotide synthesis. macroH2A1 was also found to rewire lipid and carbohydrate metabolism in HCC toward LCSCs (Lo Re et al., 2018a).

#### REX1

REX1 is also called zinc finger protein 42 (ZFP42) (Jiang et al., 2002) and has been studied in multiple cancer types (Kim et al., 2011). Steve TLUK et al. found that REX1 was frequently downregulated in HCC tumors. Furthermore, they explored the possibility that REX1 silencing was regulated by promoter hypermethylation, histone methylation and histone acetylation in human HCC. In addition, silencing of REX1 potentiated the tumorigenesis and metastasis potential of HCC. The molecular mechanism by which REX1 deficiency enhanced the stemness appeared to involve p38 MAPK signaling regulation in a mitogen-activated protein kinase kinase 6 (MKK6)-dependent manner (Luk et al., 2019). Furthermore, REX1 silencing promoted F-actin reorganization and changed oxidative stress levels through a p38 MAPK-dependent pathway.

#### MYCN

MYCN is a member of the MYC family, which comprises basic helix–loop–helix–zipper transcription factors. MYCN is one of the central regulators of the growth-promoting signal transduction that maintains stem-like properties (Takahashi and Yamanaka, 2006). Acyclic retinoid (ACR) is capable of preventing HCC recurrence in hepatitis C virus (HCV)-positive patients who have undergone curative removal of primary tumors (Muto et al., 1996). Recent research found that ACR significantly inhibited MYCN expression at both the gene and protein level. Mechanistically, MYCN is expressed at high levels in S and G2 phases in cells. Knockdown of MYCN repressed cell cycle progression and induced cell death. Furthermore, MYCN expression was correlated with EpCAM, Alpha-fetoprotein (AFP), and CD133 expression and activated Wnt/β-catenin signaling in HCC (Qin et al., 2018).

#### ZFX

Zinc finger protein X-linked (ZFX) is a zinc finger transcription factor encoded on the mammalian X chromosome and is frequently upregulated in various malignancies (Jiang and Liu, 2015; Li Y. et al., 2015). One study demonstrated that high ZFX expression conferred self-renewal and chemoresistance properties to HCC cells by binding of the SRY-box transcription factor (Sox)2 and Nanog (Lai et al., 2014). Recently, Chao Wang et al., reported that ZFX expression in LCSCs was relevant to poor prognosis. Consistently, silencing ZFX expression suppressed tumorigenicity and the metastatic potential of EpCAM^+^ LCSCs *in vitro*. Interestingly, knockdown of ZFX suppressed the expression of several β-catenin target genes, such as cyclin D1, c-Jun and c-Myc. More importantly, ZFX was responsible for maintaining stem-like features of EpCAM^+^ LCSCs by facilitating β-catenin nuclear translocation and transactivation (Wang C. et al., 2017).

#### HOXB7

Homeobox B7 (HOXB7) belongs to the homeobox gene family, which plays a role in some solid tumors (Chile et al., 2013; Joo et al., 2016). EMT causes epithelial cells to lose their cell-cell adhesions, plays an important role in HCC metastasis (Candini et al., 2015). A previous study showed that HOXB7 enhanced the proliferation and self-renewal of LCSCs (Care et al., 1999). A recent investigation showed that HOXB7 was highly expressed in HCC cells and could facilitate growth and metastasis of cell stemness and EMT, correlating with poor prognosis. Further mechanistic research suggested that HOXB7 promoted metastasis by activating the Akt pathway to upregulate c-Myc and Slug in HCC. In conclusion, HOXB7 promotes EMT and modulates the PI3K/Akt/c-Myc axis to facilitate stem cell pluripotency in HCC (Huan et al., 2017).

#### Tcf7l1

The β-catenin-transcription factor 7 like 1 (Tcf7l1) shows high expression in many malignant tumors and has a crucial effect on the Wnt/β-catenin pathway (Murphy et al., 2016). However, another study reported the opposite results, finding that Tcf7l1 expression was down-regulated in LCSCs and associated with poor survival of HCC patients. Further mechanistic research showed that Tcf7l1 attenuation upregulated the expression of stemness genes, including kruppel like factor (KLF)4, OCT4 and Nanog, and down-regulated the expression of differentiation genes, including glucose-6-phosphatase (G6p), albumin and transthyretin. Tcf7l1 knockdown further impacted the protein expression of Nanog. Moreover, Tcf7l1 phosphorylation and protein degradation through the mitogen-activated protein kinase (MEK)/extracellular signal regulated kinase (ERK) pathway were negatively regulated by extracellular insulin-like growth factor (IGF) signaling (Shan et al., 2019).

Furthermore, Sox12 is a potential marker in LCSCs (Zou et al., 2017). Another a transcription factor, E26 transformation-specific transcription factor ELK3 (ELK3), is activated by mitogen-activated protein kinase-associated signaling pathways (Buchwalter et al., 2005). The expression of ELK3 was upregulated in CD133^+^/CD44^+^ HCC cells. Furthermore, silencing the expression of ELK3 in CD133^+^/CD44^+^ LCSCs could downregulate their metastatic potential by modulating hypoxia inducible factor 1α (HIF-1α) expression (Lee J.H. et al., 2017). In addition, forkhead box M1 (FOXM1) belongs to the forkhead box protein family, which plays an important role in DNA replication, mitosis and genomic stability (Laoukili et al., 2005). FOXM1 inhibited LCSC proliferation, migration, invasion, colony formation and EMT by promoting apoptosis. Furthermore, silencing of FOXM1 suppressed the expression of Sox2, OCT4, and Nanog in LCSCs by decreasing the expression of acetaldehyde dehydrogenase-2 (Chen et al., 2019). In addition, ring finger protein 1 (Ring1), an essential cofactor of polycomb group proteins, was upregulated in HCC and targeted p53 to promote cancer cell proliferation (Xiong et al., 2015; Shen et al., 2018). Zhu et al. (2019) found that overexpression of Ring1 activated the Wnt/β-catenin signaling pathway and drove malignant transformation of LCSCs. In addition, KLF8, which belongs to the KLF family of transcription factors (Pearson et al., 2008), is highly expressed in LCSCs, and KLF8 gene silencing suppressed the invasion and migration of LCSCs. For the further mechanism, Wnt/β-catenin signaling participates in the KLF8 regulation process (Shen et al., 2017).

### Oncogenes or Oncoproteins Mainly Located in Both the Nucleus and Cytoplasm of Human Cells

#### Shp2

Src-homology 2 domain–containing phosphatase 2 (Shp2) is a non-receptor protein tyrosine phosphatase encoded by PTPN11 (Feng et al., 1993). Studies have demonstrated that Shp2 highly expression is associated with poor prognosis in various malignancies (Aceto et al., 2012; Han et al., 2015). A recent study found that upregulation of Shp2 facilitated expansion by promoting self-renewal of LCSCs. Further research on the mechanism revealed that Shp2 dephosphorylated cell division control protein 73 in the cytosol of hepatoma cells and that Shp2 could augment nuclear accumulation of β-catenin. Furthermore, Shp2 increased β-catenin accumulation by inhibiting the glycogen synthase kinase GSK3β in LCSCs (Xiang et al., 2017).

#### ZIC2

Zic family member 2 (ZIC2) belongs to the zinc finger transcription factor gene family (Benedyk et al., 1994). A previous study showed that ZIC2 was enhanced in various tumors and regulated tumorigenesis (Marchini et al., 2012). Bromodomain PHD finger transcription factor (BPTF) is the largest subset of the nuclear remodeling factor (NURF) chromatin remodeling complex (Li et al., 2006). The NURF complex is responsible for embryonic differentiation, development and stemness maintenance (Cherry and Matunis, 2010). A recent study demonstrated that ZIC2 expression was high in LCSCs and could regulate their self-renewal. Mechanistically, ZIC2 can bind to the upstream region of OCT4 and initiate its activation. Importantly, ZIC2 interacts with the NURF complex in the nucleus of HCCs. Furthermore, ZIC2 silencing abolished its binding capacity to the NURF complex, but depletion of the NURF complex did not affect the binding capacity of ZIC2 to the OCT4 promoter. These findings suggest that the NURF complex regulates OCT4 expression directly. In sum, ZIC2 can sustain the stemness of LCSCs by recruiting the NURF complex to trigger OCT4 activation (Zhu P. et al., 2015).

#### BPTF

The NURF complex can also modulate chromatin structure by targeting genes that make transcription factors more accessible (Song et al., 2009). One study reported that BPTF could activate oncogenic signaling and synergize with other proteins to regulate tumor progression (Dar et al., 2016; Richart et al., 2016). Another recent study reported high BPTF expression in HCC. In addition, down-regulation of BPTF expression affected cell colony formation, proliferation, chemotherapy resistance and apoptosis and tumor progression in HCC. However, human telomerase reverse transcriptase (hTERT), a catalytic subset of the telomerase holoenzyme complex, synthesizes telomeres using its own RNA as a template and then adds the telomeres to the ends of chromosomes (Liu N. et al., 2016). Further study of the molecular mechanisms showed that BPTF promotes tumor cell proliferation, tumor metastasis and stemness maintenance by activating hTERT expression in HCCs (Zhao et al., 2019).

#### IRAK1

Interleukin-1 receptor-associated kinase 1 (IRAK1) phosphorylation is implicated in tumorigenesis (Dussiau et al., 2015). However, the role of IRAK1 itself in TICs and HCC is not clear. In a recent study, Cheng et al. (2018) found that overexpression of IRAK1 in HCC was related to poor prognosis. Importantly, IRAK1 was found to regulate self-renewal, tumorigenicity, chemoresistance and TIC expression in HCC. Mechanistically, knockdown of IRAK1 revealed that Aldo-Keto Reductase Family 1 Member 10 (AKR1B10) was a target of IRAK1 mediated through activator protein 1 (AP-1) activation. More importantly, IRAK1 augmented stemness and chemoresistance through AP-1/AKR1B10 signaling in HCC (Cheng et al., 2018).

#### BORIS

BORIS is the paralog of CCCTC-binding factor (CTCF), also called CCCTC-binding factor-like (CTCFL) (Marshall et al., 2014). Notably, increasing evidence shows that BORIS is expressed in CSCs and associated with CSC-like properties (Alberti et al., 2014, 2015). In one study, Liu et al. (2017) found that BORIS overexpression increased CD90 expression, drug resistance, migration, invasion and stem cell marker (Sox2, OCT4, and c-Myc) expression in human HCC cells. Mechanistically, BORIS regulates OCT4 via epigenetic modification, with changes in the histone methylation status of the OCT4 promoter at CTCF sites. BORIS maintains an active chromatin conformation via increasing the histone 3 lysine 4 bimethylation (H3K4me2)/histone H3 lysine 27 trimethylation (H3K27me3) ratio to enhance OCT4 expression (Liu et al., 2017).

#### TARBP2

Transactivation response element RNA-binding protein 2 (TARBP2) is a double-stranded RNA-binding protein governing the translation of mRNA (Gatignol et al., 1991). TARBP2 was suggested to be a potential regulatory factor in CSCs (De Vito et al., 2012). The study identified that restoration of TARBP2 expression resensitized HCC to sorafenib. TARBP2-mediated sensitization of HCC to sorafenib was miRNA-independent. Interestingly, TARBP2 protein was destabilized by autophagic-lysosomal proteolytic degradation in HCC cells. Mechanistically, downregulated TARBP2 expression promoted sorafenib resistance via stabilization of Nanog expression and increased LCSC properties in HCC cells (Lai et al., 2019).

### Oncogenes or Oncoproteins Mainly Located in the Cytoplasm of Human Cells

#### iNOS

An increasing number of studies suggest that NO, which is produced by inducible NO synthase (iNOS), promotes tumor initiation (Granados-Principal et al., 2015; Davila-Gonzalez et al., 2017). Additionally, the Notch signaling pathway can promote CSC self-renewal, migration, differentiation, proliferation and survival in several malignancies (Androutsellis-Theotokis et al., 2006). A recent study reported that iNOS exhibited high expression in CD24^+^/CD133^+^ LCSCs. Furthermore, iNOS/NO was associated with aggressive human HCC by activating the Notch signaling pathway. The Notch signaling activation was dependent on upregulation of iRhom-2 and 3′,5′-cyclic guanosine monophosphate (cGMP)/protein kinase G (PKG)-mediated activation of transarterial chemoembolization (TACE). These studies provide a mechanism explaining the tumorigenic effects of iNOS in LCSCs and indicate that targeting iNOS could have therapeutic benefits in HCC (Wang et al., 2018).

#### GLS1

Glutaminase 1 (GLS1), which converts glutamine to glutamate, is associated with proliferation, growth and metabolism in cancer cells (Aledo et al., 2000). Previous studies have demonstrated that GLS1 is responsible for cell invasion and migration, which predict a poor prognosis in HCC (Yu et al., 2015). GLS1 mRNA has been reported to generate two isoforms, with the shorter form named glucose absorption capacity (GAC) and the longer form called α-ketoglutaric acid (KGA) (Elgadi et al., 1999). In a recent report, Yitao Ding et al., reported that both the KGA and GAC isoforms were exclusively located in the mitochondrial matrix. In addition, the mitochondrial matrix protein GLS1 is highly expressed in LCSCs. Mechanistically, targeting GLS1 or glutamine metabolism increased reactive oxygen species (ROS) accumulation, which suppressed β-catenin translocation from the cytoplasm to the nucleus, leading to a decrease in stemness-related gene expression. GLS1 regulates the stemness features of LCSCs via ROS/Wnt/β-catenin signaling (Li B. et al., 2019).

#### KIF15

Kinesin family member 15 (KIF15) plays an important role in many malignant tumors with a tetrameric spindle motor structure (Reinemann et al., 2017; Sheng et al., 2019). Nevertheless, the mechanism by which KIF15 targets LCSCs remains unclear. A recent study found that KIF15 was highly expressed in HCC tissues from patients with higher recurrence and shorter overall survival. Experimentally, low ROS levels in the tumor microenvironment have been verified to support the stemness of CSCs (Lee K.M. et al., 2017). KIF15 can promote LCSC stemness. Further mechanistic research showed that KIF15 markedly decreased intracellular ROS levels and increased the LCSC phenotype via phosphoglycerate dehydrogenase (PHGDH). Furthermore, the chromatin-associated protein ANCCA (also known as ATAD2, the ATPase family AAA domain-containing protein 2) appears to have an important role in enhancing KIF15 expression (Li Q. et al., 2019).

#### ANXA3

Annexin A3 (ANXA3), which belongs to the annexin family of Ca2^+^-dependent phospholipid-binding proteins, has the ability to promote tumorigenesis and resistance to chemotherapy (Raynal and Pollard, 1994; Pan et al., 2015). Stephanie Ma et al., found that high expression of both secretory and endogenous ANXA3 was correlated with HCC pathogenesis. They further found that secretory ANXA3 could be detected in sera of HCC patients and that the secretory ANXA3 played a crucial role in maintenance of LCSC-like properties. Mechanistically, exogenous ANXA3 was internalized via caveolin-1-dependent endocytosis. In addition, exogenous ANXA3 overexpression resulted in c-Jun N-terminal kinase (JNK) pathway activation, as evidenced by increased c-Myc expression, reduced p21 expression and increased JNK activity. In sum, ANXA3 is responsible for enhancing stemness in CD133^+^ LCSCs via the JNK pathway (Tong et al., 2015).

#### Cygb

Cytoglobin (Cygb) is a member of the human hexacoordinate hemoglobin family. Cygb is a tumor suppressor whose deficiency contributes to tumor recurrence and poor prognosis in multiple malignancies (Xu et al., 2013; Thuy le et al., 2016). Oxidative-nitrosative stress (ONS) is an independent etiologic factor in HCC tumorigenesis (Wang Z. et al., 2016). Accumulating evidence indicates that the interaction of ONS with CSCs promotes tumorigenesis, progression, and hemoradiotherapy resistance (Su et al., 2016). A recent study found that Cygb was deregulated in HCC tissue and the decrease aggravated the growth of LCSCs. Furthermore, Cygb absence promoted LCSC phenotypes and PI3K/Akt activation in HCC progression but inhibited HCC proliferation and LCSC stemness in an ONS-dependent manner (Zhang et al., 2019).

### Oncogenes or Oncoproteins Mainly Located in the Cell Membrane in Human Cells

#### NUMB

NUMB is a tumor suppressor and cell fate determinant, and loss of NUMB expression has been observed in cancer (Colaluca et al., 2008). The p53-NUMB complex was independently demonstrated to be a tumor suppressor (March et al., 2011). Recently, study showed that NUMB phosphorylation plays a crucial role in tumor-initiating cell self-renewal and liver tumorigenesis via the Nanog pathway. Further mechanistic research, Nanog increased phosphorylation of NUMB and decreased p53 by modulating the atypical protein kinase C zeta/Aurora A kinase (aPKCf-AURKA) pathway, which is an upstream pathway for NUMB phosphorylation. Furthermore, the phosphorylation of NUMB by Nanog destabilized the NUMB-p53 complex, leading to destabilization of p53 and subsequent high self-renewal of TICs (Siddique et al., 2015).

#### AQP3

Aquaporin 3 (AQP3) is a member of the water channel protein family, which can be found in the plasma membranes of various cells (Verkman, 2012). Studies have shown that aberrant AQP3 expression contributes to several malignant tumors (Huang X. et al., 2017; Xiong et al., 2017). Accumulating evidence supports the notion that AQP3 is related to maintain of CSC stemness (Zhou et al., 2016). Recently, Yawei Wang and his colleagues reported that AQP3 expression was high in HCCs. Additionally, depletion of AQP3 suppressed the proliferation and invasion of CD133^+^ HCC. In addition, AQP3 promoted LCSC properties by regulating STAT3 nuclear translocation and phosphorylation (Wang et al., 2019a).

#### ITGA7

Integrins are a subclass of glycoproteins that mediate cell-cell or cell-extracellular adhesion (LaFlamme et al., 2018). Integrin alpha 7 (ITGA7) was demonstrated to maintain stemness through targeting CSC biomarkers in various cancers (Ming et al., 2016). Recently, Ge et al. (2019) found that knockdown of ITGA7 suppressed proliferation, reduced CSC marker expression levels (CD44, CD133, and OCT4) and enhanced apoptosis by targeting the protein tyrosine kinase 2 (PTK2)-PI3K-Akt signaling pathway in liver cancer cells. However, overexpression of ITGA7 promoted proliferation and suppressed apoptosis but not CSC marker expression via the PTK2-PI3K-Akt signaling pathway. Then, they further performed compensation experiments, which verified that ITGA7 regulates cell stemness through the PTK2-PI3K-Akt signaling pathway (Ge et al., 2019).

#### CD44s, CLDN1, and FZD2

Some oncogenes are located in cell junctions, the cell membrane, and the basolateral cell membrane and have a common mechanism for targeting EMT. Increasing evidence suggests that EMT is connected with CSC properties and cancer metastasis and recurrence (Choi and Diehl, 2009). A previous study reported that the isoform switch to CD44s was essential for cells to undergo EMT (Brown et al., 2011). Recently, Asai et al. (2019) investigated the roles of CD44s in LCSCs. Knockdown of CD44s expression resulted in decreased spheroid formation and increased drug sensitivity. In addition, another study reported that CD44s is involved in maintenance of LCSCs via the notch receptor 3 (NOTCH3) signaling pathway (Asai et al., 2019). Moreover, claudin 1 (CLDN1) plays a critical role in the EMT process in HCC (Suh et al., 2017). However, transmembrane protease serine 4 (TMPRSS4) is a contributing mediator during EMT and an inducer of the CSC phenotype in multiple tumors (Huang et al., 2014; de Aberasturi et al., 2016). Mahati et al. (2017) observed that TMPRSS4 and CLDN1 were remarkably upregulated in HCC tissues, while overexpression of CLDN1 induced EMT and CSC behaviors via TMPRSS4 in HCC. Mechanistically, Ou et al. (2019) provided evidence that Frizzled 2 (FZD2) is a driver of EMT and CSC properties in HCC.

### Oncogenes or Oncoproteins in Other Locations or Pathways in Human Cells

#### RACK1, Tg737, and MAGE-A9

Some oncogenes are located in many parts of the cell, for example, the cell membrane, cytoplasm, cytoskeleton, perinuclear region, nucleus, cell projections, dendrites, and phagocytic cups. In the same manner, they can target different targets and ultimately affect the stemness of LCSCs. Receptor for activated C kinase 1 (RACK1) belongs to the Trp-Asp repeat protein family and is an adaptor protein involved in multiple signaling pathways (Bourd-Boittin et al., 2008). Overexpression of RACK1 is associated with short overall survival and a high recurrence rate in HCC (Ruan et al., 2012). In recent work, RACK1 was found to directly stabilize Nanog, thus contributing to the self-renewal and chemoresistance of LCSCs (Cao et al., 2019). In addition, the Tg737 gene is a mouse intra-flagellar transport 88 homologue that was first identified in Chlamydomonas (Pazour et al., 2000). Previous studies have shown that Tg737 expression highly suppresses LCSC properties. Consistently, Tg737 gene silencing was significantly associated with tumor differentiation, metastasis, and invasion and alpha-fetoprotein levels (You et al., 2017). Furthermore, knockdown of Tg737 caused liver cancer cells to acquire LCSC properties during malignant transformation, because Tg737 regulated a double-negative feedback loop between Wnt/β-catenin and hepatocyte nuclear factor 4-alpha, resulting in EMT (Huang Q. et al., 2017). Moreover, the melanoma antigen gene (MAGE) family represents one of the largest groups of human tumor-associated antigens. MAGE-A9, a member of the MAGE-A gene family, is frequently expressed in various human tumors (Gu et al., 2014). MAGE-A9 contributes to malignant biological phenotypes, including cell proliferation, chemoresistance and migration of EpCAM^+^ HCC cells (Wei et al., 2018).

#### OPN, CCN3, and LOX

A subset of secretory oncogenes can localize in many parts of the cell. Osteopontin (OPN) is a subclass of phosphorylated glycoproteins and is associated with chemoresistance in many malignant tumors (Pang et al., 2011; Hsieh et al., 2013). Considerable evidence has revealed that OPN enhances the CSC phenotype in cancer (Pietras et al., 2014). Guoke Liu et al., reported that secreted OPN induced autophagy by sustaining forkhead box O3a (FoxO3a) stability and binding with its integrin. The autophagy promoted LCSC properties and chemoresistance (Liu G. et al., 2016). Another study found that down-regulation of OPN expression in CD133^+^/CD44^+^ cells suppressed migration and proliferation by regulating DNA methyltransferase (DNMT)1 expression. Downregulation of DNMT1 expression reduced global DNA methylation. Additionally, various levels of OPN exhibited different sensitivities to 5 Aza (Gao et al., 2018). Moreover, cellular communication network factor 3 (CCN3) is associated with the malignant phenotype of HCC. Furthermore, one study found that CCN3 overexpression enhanced survival and increased in vivo metastasis of HCC. Mechanically, CCN3 affects the upregulation of OPN and coagulation factors, which led to enhance stemness of LCSCs (Jia et al., 2017). Lysyl oxidase (LOX) is a secreted enzyme, that contributes to regulation of various factors, including extracellular matrix (ECM) maintenance, migration and angiogenesis (Zhu J. et al., 2015; Ribeiro et al., 2017). A recent study revealed that LOX gene expression was upregulated in cell spheres and led to more vascular enrichment in a mouse xenograft model. Furthermore, LOX expression increased vascular endothelial growth factor (VEGF) and enhanced the tube formation capacity of endothelial cells. These findings provide a novel mechanism of LOX in regulation of TICs in HCC (Yang et al., 2019).

## Conclusion

Hepatocellular carcinoma is a solid cancer with high morbidity and mortality. Evidence has shown that the existence of LCSCs can contribute to HCC tumor initiation, drug resistance, metastasis and recurrence. Intriguingly, LCSC elimination seems to be an ideal method to defeat HCC. Therefore, specific targeting of LCSCs may repress the malignant biological behaviors of HCC and improve curative effects. Mounting data have suggested that LCSCs develop through a multistep process associated with RNAs, genes, proteins, pathways, factors, autophagy, the microenvironment and the networks between them. Thus, a better understanding of the molecular mechanisms underlying HCC initiation and progression is a pressing requirement. Additional studies are urgently necessary to facilitate exploration of new therapeutic targets and effective treatment strategies. Through classification of the studies on LCSC targeting published in the past 5 years, we found that most of the studies focused on ncRNAs (especially the miRNAs and lncRNAs), oncogenes, oncoproteins and the crosstalk between their upstream/downstream genes and molecular pathways.

miRNA is a major class of non-protein-coding transcripts that instead function in posttranscriptional regulation of genes. Several miRNAs can enhance LCSC features, and opposite effects can be found with other miRNAs. lncRNAs regulate gene expression through different ways, such as protein and miRNAs networks. In this review, we observed that a number of miRNAs and lncRNAs might serve as novel markers or provide potential therapeutic targets in LCSCs. Dysregulation of miRNAs or lncRNAs could be used to identify and characterize LCSCs based on their interaction with pivotal signaling pathways, focusing on the Wnt/β-Catenin signaling pathway (such as miR-1246, miR-429, Lnc-β-Catm, lncTCF7, and CUDR), IL6/JAK2/STAT3 signaling pathway (such as miR-500a-3p, miR-589-5p, DLX6-AS1, and Lnc-DILC), PI3K/Akt/Bad signaling pathway (such as miR302a/d, miR-1305, miR24-2, miR-25, and lncRNA-HULC) and certain genes, including OCT4 (such as miR-1246 and miR-429) and Nanog (such as miR24-2), as well as on cell surface proteins or cellular prognostic markers that have been identified to be characteristic of LCSCs, such as EpCAM (miR26b-5p and miR-429).

Genes support the basic structure and properties of life through their genetic effects. In this review, according to Supplementary Table 1, which presents impact factors, we found that the oncogenes and oncoproteins reported by high-impact factor studies to target LCSCs are primarily located in the nucleus and cytoplasm. Similarly, we found that many oncogenes and oncoproteins are novel potential LCSC markers located in the cell membrane or are subsecretory types. Moreover, some of the molecular mechanisms of the oncogenes or oncoproteins that target LCSCs are the same and involve several key pathways, including the Wnt/β-catenin signaling pathway (such as Sox9, Shp2, MYCN, ZFX, GLS1, Tg737, KLF8, and Ring1), Notch signaling pathway (such as iNOS and CD44s), PI3K/Akt/c-Myc pathway (such as HOXB7, Cygb, and ITGA7) and STAT3 pathway (such as AQP3). The target genes include Nanog (such as Sox9, NUMB, TARBP2, RACK1, and FOXM1) and OCT4 (such as Sox9, ZIC2, FOXM1, and BORIS), along with LCSC biomarkers (such as CD133, CD44, and EpCAM).

From the above observations, in addition to LCSC surface biomarkers, we emphasize the role of three signaling pathways and two genes that influence LCSCs. The first is the Wnt/β-catenin signaling pathway. The Wnt/β-catenin signaling pathway has been identified as one of the most frequent participants in CSCs (Fodde and Brabletz, 2007). Dramatically, the Wnt/β-catenin signaling pathway, which involves translocation of β-catenin to the nucleus, is heavily implicated in LCSCs (Yamashita et al., 2007). Moreover, the final nuclear transfer can induce transcription of prominent targets, such as c-Myc (He et al., 1998) and CD44 (Wielenga et al., 1999). CD44 has also been identified as a biomarker of LCSCs (Zhu et al., 2010). In addition, EpCAM is a direct transcriptional target of the Wnt/β-catenin signaling pathway in HCCs (Yamashita et al., 2007).

The second signaling pathway is the PI3K/Akt/c-Myc pathway. PI3K-Akt has been shown to promote cancer stemness in various cancer types (Hambardzumyan et al., 2008; Bleau et al., 2009). Elevated phosphatidylinositol 3,4,5-trisphosphate (PIP)3 levels lead to activation of multiple kinases, including phosphoinositide-dependent protein kinase 1 (PDK1), which phosphorylates downstream targets, such as Akt. Activated Akt phosphorylates numerous substrates to regulate vital cellular processes, including tuberous sclerosis complex 2 (TSC2), NF-κB and GSK3β (Vanhaesebroeck et al., 2010). Furthermore, the PI3K-Akt pathway has been reported to augment the expression of c-Myc (Tsai et al., 2012; Zhang H.F. et al., 2016). Interestingly, one study demonstrated synergistic interactions of CD44 and TGF-β1 in EMT induction via the Akt/GSK-3β/β-catenin pathway in HCCs (Park et al., 2016). Here, we found that c-Myc is a coactive gene in the Wnt/β-catenin signaling pathway and PI3K/Akt signaling pathway. Interestingly, the proto-oncogene Myc is the frequent event in many cancers (Soucek et al., 2008). Myc can be activated via Wnt/β-catenin, PI3K/Akt, MAPK/extracellular signal-regulated kinase (ERK) and Hedgehog. Mechanically, the activated Myc gene affects target genes mediation including chromatin remodeling and DNA-methylation (Sridharan et al., 2009).

The third signaling pathway is the IL6/JAK2/STAT3 signaling pathway. IL-6 produced by tumor-associated macrophages (TAMs) can activate the STAT3 signaling pathway to promote CD44^+^ LCSCs (Wan et al., 2014). Therefore, an IL-6 receptor blocking antibody (such as tocilizumab) is a novel therapeutic strategy for targeting LCSCs. Simultaneously, it has been demonstrated that targeting of the TGF-β pathway using indirect modulation of IL6/STAT3 appears to effectively eradicate LCSC features (Lin et al., 2009).

OCT4, which belongs to the POU family, is the most important stem cell factor and is considered the master regulator in the maintenance of stem cell potency (Nichols et al., 1998). Active OCT4 can directly regulate two downstream stem cell regulator genes, Nanog and SOX2, promoting LCSC-like phenotypes (Babaie et al., 2007). Many studies have identified that there is a correlation between OCT4 and LCSCs (Murakami et al., 2015). Nanog has been proposed as an important regulator modulating the phenotype of CSCs in various of cancer types (Shan et al., 2012; Chen et al., 2016). Furthermore, one study has reported that overexpression of CD24 is accompanied by increased STAT3 and Src activities (Bretz et al., 2012). Interestingly, STAT3-mediated Nanog expression can regulate self-renewal and tumor initiation in CD24^+^ LCSCs (Lee et al., 2011).

Altogether, non-coding RNAs, genes, and signaling pathways form a network that affects the characteristics of LCSCs. Targeting LCSCs via ncRNAs, oncogenes, oncoproteins or signaling pathways holds promise for preventing disease relapse. In addition, some small molecular agents have been studied extensively. However, there is still no available US FDA-approved drug that is likely to be clinically effective for HCC. It is now clear that all RNAs, genes, proteins and signaling pathways function as a coordinated network rather than operating in isolation. Thus, we should find a key node in the LCSC network. In this review, we summarize three pathways: the Wnt/β-catenin pathway, PI3K/Akt pathway, and IL6/JAK2/STAT3 pathway and their targeting gene c-Myc. Furthermore, we conclude that two important genes are OCT4 and Nanog. They play a pivotal role in LCSC regulation and HCC prognosis. There is a potential opportunity to achieve great therapeutic effects by targeting the above signaling pathways or genes in LCSCs. However, their dual oncogenic and biological functions indicate that targeting should be conducted with caution.

## Author Contributions

YZ conceived and designed the work and approved the final version. JL acquired the data and wrote the manuscript. Both authors read and approved the final manuscript.

## Conflict of Interest

The authors declare that the research was conducted in the absence of any commercial or financial relationships that could be construed as a potential conflict of interest.

## Footnotes

1 https://www.genecards.org/
