# Supplementary material for: Recent Advances in Liver Cancer Stem Cells: Non-coding RNAs, Oncogenes and Oncoproteins
Source: Front Cell Dev Biol. 2020 Oct 7;8:548335. doi: 10.3389/fcell.2020.548335 (PMC7575754; doi:10.3389/fcell.2020.548335)
Supplement: Supplementary file 1 [file Data_Sheet_1.docx]

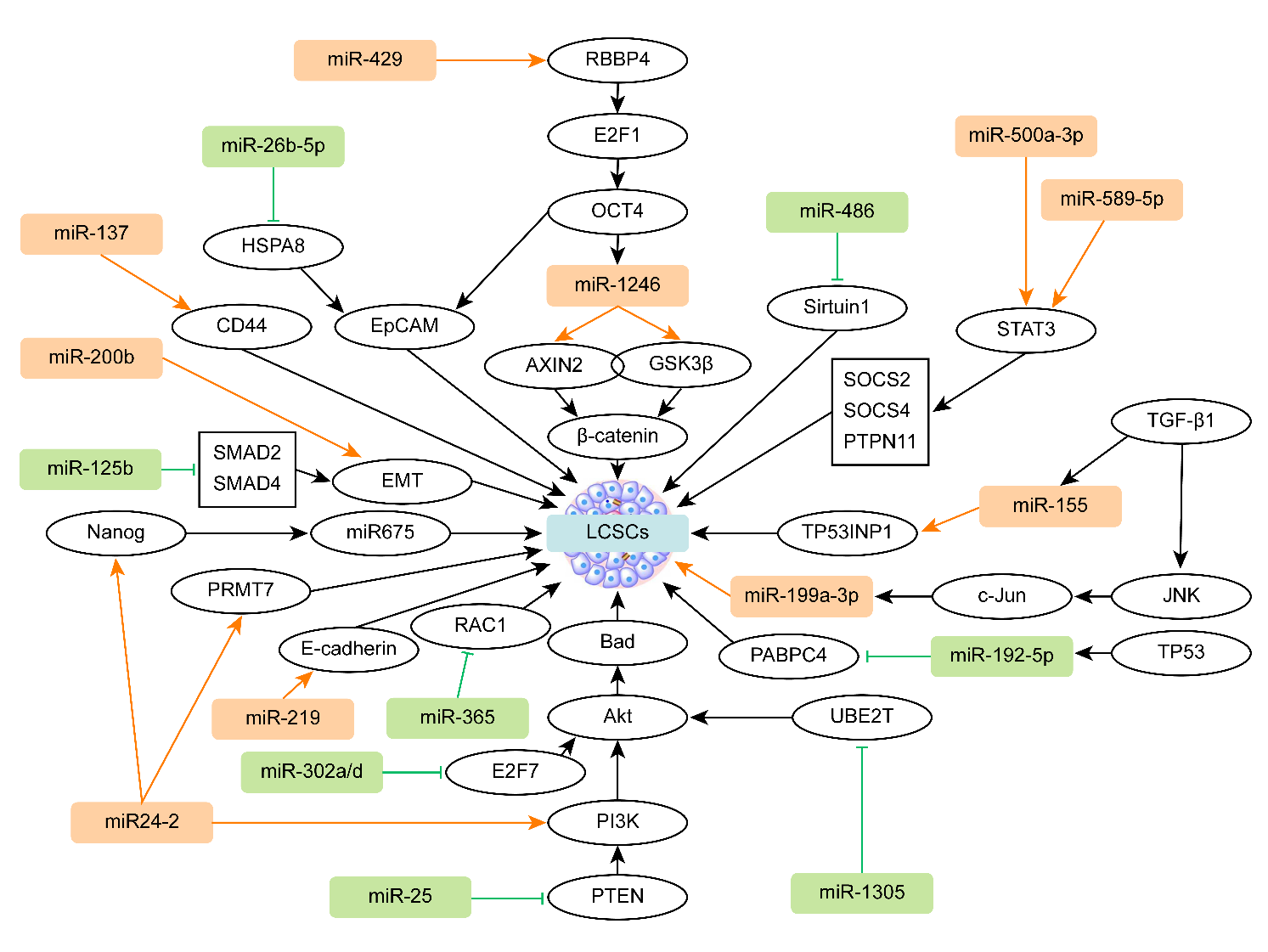


**Supplementary Figure 1**. miRNAs associated with LCSCs. Dysregulation of miRNA expression has been linked to LCSC self-renewal, invasion, migration and drug resistance. In the figure, orange indicates miRNAs whose overexpression enhances LCSC properties, and green indicates miRNAs whose overexpression suppresses LCSC properties.


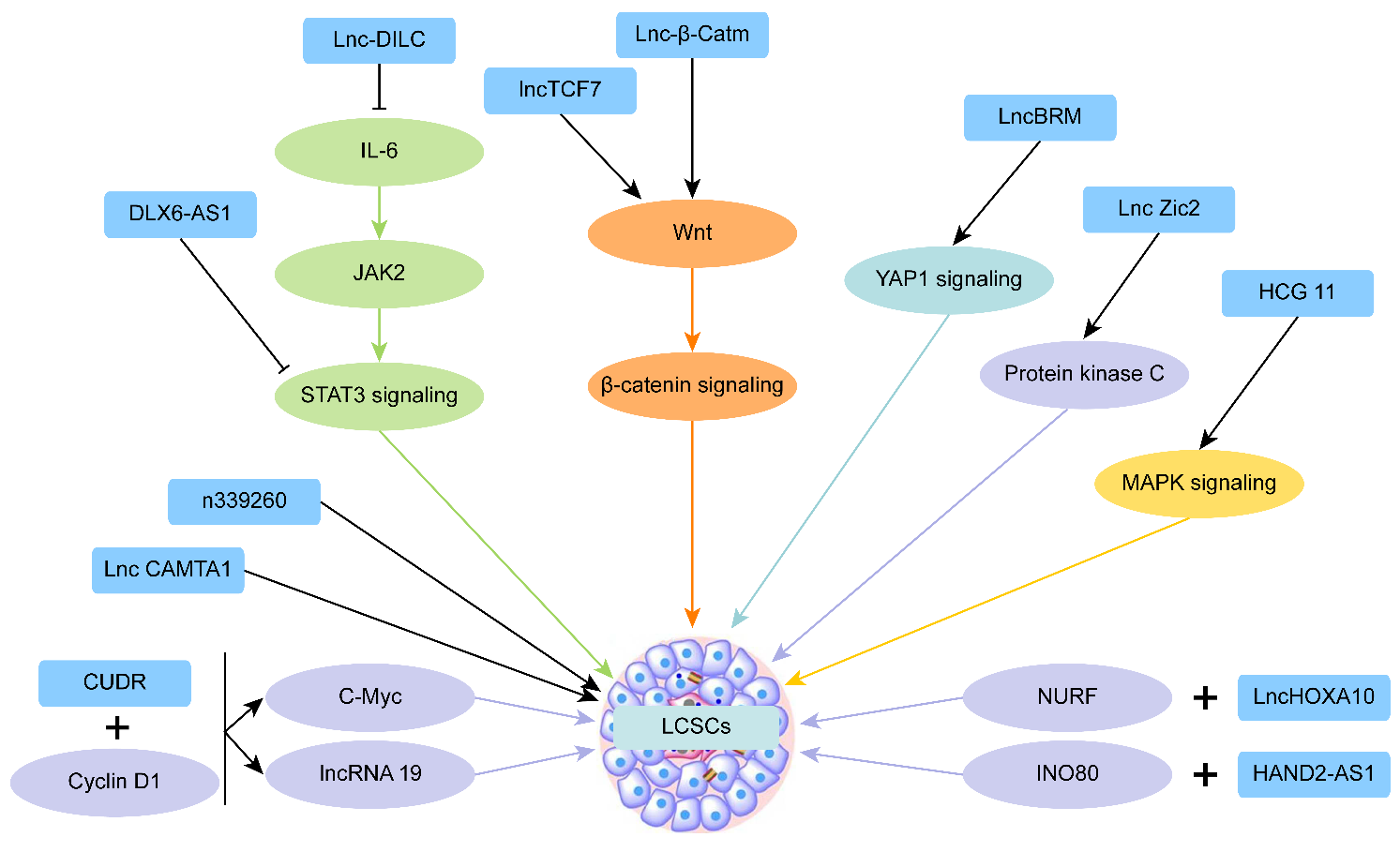


**Supplementary Figure 2**. lncRNAs associated with LCSCs. Dysregulation of lncRNA expression has been associated with the biological function of LCSCs. In the figure, blue represents various dysregulated lncRNAs, and the other colours indicate lncRNAs that act on LCSCs in different ways. Among them, lnc-DILC and DLX6-AS1 play suppressive roles, while the others have promotive effects on LCSC properties.

# Supplementary Material

**Table 1. The role of oncogenes or oncoproteins associated with LCSCs**

| **Impact Factor** | **Oncogenes** | **Location in Human** | | **Characteristics** | **Mechanism** | **References** |
| --- | --- | --- | --- | --- | --- | --- |
| **>10 points** | **Sox9** | | Nucleus | a transcription factor | Reduced stem cell transcription factors BMI-1, OCT4, Nanog, α-fetoprotein and β-catenin. | (Liu et al., 2016a) |
|  | **MacroH2A1** | | Nucleus | histone variant of the H2A histone family | Regulated by a phosphorylated nuclear factor kappa B p65 pathway; showed two changes in glucose and lipid metabolism | (Lo Re et al., 2018a; Lo Re et al., 2018b) |
|  | **REX1** | | Nucleus | called zinc finger protein 42 | P38 MAPK pathway and MKK6 binding. | (Luk et al., 2019) |
|  | **Shp2** | | Nucleus/Cytoplasm | a nonreceptor protein tyrosine phosphatase | β-catenin signaling. | (Xiang et al., 2017) |
|  | **ZIC2** | | Nucleus/Cytoplasm | a zinc finger transcription factor | recruited the NURF complex to trigger OCT4 activation. | (Zhu et al., 2015b) |
|  | **NUMB** | | Membrane | a tumor suppressor and cell fate determinant, association with p53 | NANOG pathway. | (Siddique et al., 2015) |
| **5-10 points** | **MYCN** | | Nucleus | MYC family of transcription factors | Repressed Cell-Cycle progression and induced cell death; correlated with EpCAM, AFP, CD133 and Wnt/β-catenin signaling. | (Qin et al., 2018) |
|  | **ZFX** | | Nucleus | zinc finger transcription factor encoded on mammalian X chromosome | Facilitated nuclear translocation and β-catenin transactivation. | (Wang et al., 2017a) |
|  | **HOXB7** | | Nucleus | homeobox gene family | promoted EMT and modulated the PI3K/AKT/c-Myc axis. | (Huan et al., 2017) |
|  | **Tcf7l1** | | Nucleus | as either a transcriptional repressor or an activator in a β-catenin-dependent or β-catenin-independent manner. | IGF/MEK/ERK Signaling | (Shan et al., 2019) |
|  | **IRAK1** | | Nucleus/Cytoplasm | Interleukin-1 receptor-associated kinase | AP-1/AKR1B10 signaling. | (Cheng et al., 2018) |
|  | **BPTF** | | Nucleus/Cytoplasm | nucleosome remodeling factor chromatin remodeling complex | Targeting telomerase reverse transcriptase. | (Zhao et al., 2019) |
|  | **BORIS** | | Nucleus/Cytoplasm | the paralog of CTCF | Regulated OCT4 gene via histone methylation modification. | (Liu et al., 2017) |
|  | **TARBP2** | | Nucleus/Cytoplasm | double-stranded RNA-binding protein | Stabilized Nanog expression. | (Lai et al., 2019) |
|  | **iNOS** | | Cytoplasm | inducible NO synthase could produce NO | TACE and Notch signaling pathway. | (Wang et al., 2018) |
|  | **GLS1** | | Cytoplasm | converts glutamine to glutamate | ROS/Wnt/β-catenin signaling. | (Li et al., 2019a) |
|  | **KIF15** | | Cytoplasm | kinesin family | Phosphoglycerate dehydrogenase-mediated intracellular reactive oxygen species imbalance. | (Li et al., 2019b) |
|  | **ANXA3** | | Cytoplasm | Ca2^+^-dependent phospholipid-binding protein | JNK pathway. | (Tong et al., 2015) |
|  | **AQP3** | | Membrane | water channel protein family | STAT3 nuclear translocation and STAT3 phosphorylation. | (Wang et al., 2019b) |
|  | **RACK1** | | Scattered distribution | Trp-Asp repeat protein family | stabilized Nanog. | (Cao et al., 2019) |
|  | **Tg737** | | Scattered distribution | mouse intra-flagellar transport 88 homologue | Wnt/β-catenin and hepatocyte nuclear factor 4-alpha. | (Huang et al., 2017a; You et al., 2017) |
|  | **OPN** | | secreted | phosphorylated glycoprotein | Sustained FoxO3a stability and bound with its integrin; regulated by the expression of DNMT1. | (Liu et al., 2016b; Gao et al., 2018) |
| **3-5 points** | **KLF8** | | Nucleus | KLF family of transcription factors | Wnt/β-catenin signaling. | (Shen et al., 2017) |
|  | **Sox12** | | Nucleus | transcription factor family member with sex-determining gene SRY | More tumor formation, chemo-resistance and metastasis in Sox12+ compared with Sox12-. | (Zou et al., 2017) |
|  | **Ring1** | | Nucleus | a ring finger protein | Wnt/β-catenin pathway. | (Zhu et al., 2019) |
|  | **FOXM1** | | Nucleus | forkhead box protein family | Suppressed Nanog, Sox2 and Oct4 expression by ALDH2. | (Chen et al., 2019) |
|  | **ELK3** | | Nucleus | a transcription factor | Modulated HIF-1α | (Lee et al., 2017a) |
|  | **Cygb** | | Cytoplasm | human hexacoordinate haemoglobin family | Promoted LCSC phenotypes and PI3K/AKT activation. | (Zhang et al., 2019) |
|  | **FZD2** | | Membrane | correlated with EMT status | Promoted clinically relevant EMT, and CD44+ stem-like properties. | (Ou et al., 2019) |
|  | **CLDN1** | | Membrane | played a critical role in EMT | Induced an EMT and CSC behaviors by TMPRSS4. | (Mahati et al., 2017) |
|  | **CD44s** | | Membrane | a standard isoform of CD44 | NOTCH3 and its target genes. | (Asai et al., 2019) |
|  | **ITGA7** | | Membrane | glycoprotein | PTK2-PI3K-Akt pathway. | (Ge et al., 2019) |
|  | **CCN3** | | secreted | the CCN family | Osteopontin and coagulation factors | (Jia et al., 2017) |
|  | **LOX** | | secreted | a secreted enzyme | Enhanced the tube formation capacity of endothelial cells. | (Yang et al., 2019) |
|  | **MAGE-A9** | | Scattered distribution | MAGE-A gene family | modulated liver cancer stem cell-like characteristics in EpCAM^+^ HCCs | (Wei et al., 2018) |
